# Preparation and Structure–Property Relationship Study of Piezoelectric–Conductive Composite Polymer Nanofiber Materials for Bone Tissue Engineering

**DOI:** 10.3390/polym16131952

**Published:** 2024-07-08

**Authors:** Zhengyang Jin, Suiyan Wei, Wenyang Jin, Bingheng Lu, Yan Xu

**Affiliations:** 1 School of Mechanical Engineering, Xinjiang University, Urumchi 830017, China; xjujxy@foxmail.com (Z.J.);; 2 The First Affiliated Hospital of Xinjiang Medical University, Urumchi 830054, China; 3 Mirco- and Nano-Technology Research Center, State Key Laboratory for Manufacturing Systems Engineering, Xi’an Jiaotong University, Xi’an 710049, China; 4 National Innovation Institute of Additive Manufacturing, Xi’an 710000, China

**Keywords:** nanofibers, electroactivity, electrospinning, bone tissue, Janus structure

## Abstract

This study aimed to develop Janus-, cross-network-, and coaxial-structured piezoelectric–conductive polymer nanofibers through electrospinning to mimic the piezoelectricity of bone and facilitate the conduction of electrical signals in bone tissue repair. These nanofibers were constructed using the piezoelectric polymer polyvinylidene fluoride, and the conductive fillers reduced graphene oxide and polypyrrole. The influence of structural features on the electroactivity of the fibers was also explored. The morphology and components of the various structural samples were characterized using SEM, TEM, and FTIR. The electroactivity of the materials was assessed with a quasi-static d33 meter and the four-probe method. The results revealed that the piezoelectric–conductive phases were successfully integrated. The Janus-structured nanofibers demonstrated the best electroactivity, with a piezoelectric constant d33 of 24.5 pC/N and conductivity of 6.78 × 10^−2^ S/m. The tensile tests and MIP measurements showed that all samples had porosity levels exceeding 70%. The tensile strength of the Janus and cross-network structures exceeded that of the periosteum (3–4 MPa), with average pore sizes of 1194.36 and 2264.46 nm, respectively. These properties indicated good mechanical performance, allowing material support while preventing fibroblast invasion. The CCK-8 and ALP tests indicated that the Janus-structured samples were biocompatible and significantly promoted the proliferation of MC3T3-E1 cells.

## 1. Introduction

Bone tissue repair and regeneration require a favorable microenvironment, and the electrical microenvironment is one of the important microenvironments. Electroactive polymers (including piezoelectric polymers and conductive polymers) are typical functional materials commonly used to construct electrical microenvironments at damaged tissues. Healthy tissue produces endogenous electrical signals that affect regeneration by activating ion channels on the plasma membrane. The production of endogenous electrical signals is impaired when a tissue is damaged. The repair and regeneration of tissues are promoted by compensating for the interrupted endogenous electrical signal in the damaged tissue, that is, transmitting the electrical stimulation (ES) to the site of the tissue damage [1,2].

To verify the influence of the piezoelectric effect on the activity of osteoblasts, Jenita Parssinen et al. [3] proved that β-polyvinylidene fluoride (PVDF) had the effect of inducing osteogenic differentiation; β-PVDF provided the necessary ES for cell growth. Cijun Shuai et al. [4] prepared PVDF/GO scaffolds, which showed better piezoelectricity compared with pure PVDF, and the results of in vitro experiments showed that PVDF/GO scaffolds induced MG-63 cell differentiation. Bikendra Maharjan et al. [5] fabricated in situ polymerized polypyrrole (PPY) nanoparticles immobilized by PCL/PPY conductive scaffolds via electrospinning. PCL/PPY exhibits good hydrophilicity, electrical conductivity, mechanical properties, and enhanced MC3T3-E1 cell adhesion, proliferation, and differentiation due to electrical stimulation. Piezoelectric polymers do not need an external power supply to generate electrical signals by converting mechanical stimuli, but most piezoelectric polymers are poor conductors, which limits the transmission of electrical signals and limits the ability to control stimulation, so surface electrodes are used to conduct piezoelectric signals [6]. Conductive polymers effectively conduct ES and regulate their chemical, electrical, and physical properties to meet the needs of biological applications [7]. However, they cannot produce an electrical signal alone. The role of conductive polymers is discounted when the generation of endogenous electrical signals in a tissue is greatly affected. Most current research focuses on the role of a single piezoelectric or a single conductive substance in bone tissue repair. In fact, conductive polymers and piezoelectric polymers can be compounded to construct a new type of composite electroactive polymer that independently generates and conducts electrical signals [8]. Cai Zhijiang et al. [9] blended the piezoelectric polymer polyhydroxybutyrate (PHB) with electrically conductive multi-walled carbon nanotubes (MWCNTs) to prepare PHB/MWCNT fibers by electrospinning. However, the material only possesses enhanced piezoelectricity, and the conductivity is still missing. Zhengnan Zhou et al. [10] prepared a PVDF/PPY piezoelectric–conductive polymer composite film. The electroactive composite film had better piezoelectric properties than pure PVDF, and the introduction of PPY made the composite coating electrically conductive. In addition, PVDF with a PPY coating can be polarized without an external electrode. Lijuan Du et al. [11] constructed a coaxially structured PEDOT/CS nanofiber for tissue regeneration, and the PEDOT/CS fiber has both piezoelectricity and electrical conductivity. A comparison of representative information from studies related to electroactive polymers with the present study is shown in Table 1.

Several of the studies mentioned above have prepared electroactive materials with different composite structures by different methods, and the structure of the materials is an important factor in determining their functions and properties. Electrospinning, which is commonly used in the preparation of fibrous nanomaterials with high porosity, has been introduced into the manufacture of advanced materials [12]. In addition to the common multi-jet co-electrospinning, coaxial electrospinning and side-by-side electrospinning are also mainstream means of multifunctional composite nanofiber preparation. The diameter, composition, shape and structure of fibers can be controlled by adjusting the solution parameters and process parameters [13]. However, there is a lack of relevant research on influencing the function of nanofibers by controlling the structure and external shape of the fibers.

In this study, our focus is to prepare an electroactive material with a biomimetic ECM structure by simulating the electrical microenvironment of bone repair through electrospinning. The optimal process parameters of the piezoelectric and conductive phases are first explored, and then electroactive polymer nanofiber materials with three common structures, including Janus, cross-network and coaxial structures, were prepared based on the optimal process parameters, and the effects of the structural differences on fiber properties were analyzed. The electroactive polymer nanofiber materials obtained in this study are capable of generating and conducting electrical signals on their own, and their good biocompatibility was verified by cytocompatibility, which is promising for their application in bone tissue engineering.

**Table 1:** Comparison of representative information from some related studies on electroactive polymers and the present study.

Ref.	Key Findings	Materials	Advantages/Limitation	Materials Type		Advantages/Limitation	Materials	Key Findings
[14]	Micro-bowl structures of PPY coating and ES have synergistic positive effects on osteoblast adhesion and differentiation.	PPY	Conducts electrical signals/inability to generate electrical signals autonomously	Conductive materials	Piezoelectric–conductive materials	Piezoelectricity and electrical conductivity, both self-generated and conductive electrical signals/Relatively complex preparation process	PVDF/RGO/PPY/PU	Janus structure of electrospun nanofibers has good electroactivity. Due to the structural features of Janus, the conductive phase of RGO/PPY enhances the piezoelectricity of the piezoelectric phase of PVDF with its piezoelectric constant d33 = 24.50 pC/N, which is higher than that of the pure PVDF fibers (d33 = 18.10 pC/N), and the conductivity of the composite fibers is unaffected with the electrical conductivity = 6.78 × 10^−2^ S/m, which is comparable to the pure conductive fiber (conductivity = 6.65 ± 0.13) × 10^−2^ S/m).
[5]	PCL/PPy exhibited enhanced MC3T3-E1 cell adhesion, proliferation, and differentiation in electrical stimulation conditions.	PLC/PPY						
[15]	The RGO/PPY/PDA/Sr scaffold can enhance adhesion and proliferation of MC3T3-E1 cells.	RGO/PPY/PDA/Sr						
[16]	Si-MNPs/PANI are biocompatible nanocomposites, and PANI and Si-MNPs provided electrical conductivity and magnetic susceptibility to the nanocomposite structures, respectively.	PANI/Si-MNPs						
[17]	Nanocomposite AgNPs/PANI supported cell adhesion and proliferation.	AgNPs/PANI						
[4]	The oxygen-containing functional group of GO forms a strong hydrogen bond with the fluorine group of PVDF, which induces the conversion of the α-phase to the β-phase. The PVDF/0.3GO scaffold had enhanced piezoelectricity compared to pure PVDF.	PVDF/GO	Autonomous generation of electrical signals/Cannot conduct electrical signals	Piezoelectric materials				
[18]	CPS self-hardened during hydration, and the microalkaline environment created during hydration facilitated the proliferation of osteoblasts and induced the formation of the β/γ phase in PVDF.	Ca-P-Si-doped PVDF						
[19]	The surface charge upregulated the polarization behavior of the macrophages and guided the polarization phenotype. And the cell response of osseointegration in the proper direction can be coordinated by controlling the surface potential.	P(VDF-TrFE)						

## 2. Experimental Section

### 2.1. Materials

PVDF (Mn = 1,000,000) was obtained from Arkema (Colombes, France). Polyurethane (PU) (Mn = 400,000) was obtained from WANHUA (Yantai, China). N, N-Dimethylformamide (DMF) was obtained from RHAWN (Shanghai, China). PPY and acetone (AC) were obtained from Merck (Darmstadt, Germany). Reduced graphene oxide (RGO) was obtained from Tanfeng Graphene Technology Co. (Suzhou, China).

### 2.2. Fabrication of Nanofiber Membranes

#### 2.2.1. Formulation of Spinning Solution for Piezoelectric Phase-PVDF

The optimization of the process parameters of PVDF based on piezoelectricity was performed in our previous study, so the optimal process parameters can be used directly to complete the preparation of PVDF piezoelectric nanofibers [20]. PVDF solution was prepared by dissolving PVDF in DMF/AC, the volume ratio of DMF to AC was 3:2, and the total polymer concentration was 15 wt.%. After continuous stirring for 4 h at room temperature, the solution was left to stand for 30 min to obtain a homogeneous solution without air bubbles.

#### 2.2.2. Optimization of Process Parameters for Conductive-Phase PU/RGO/PPY

Unlike the optimization of the piezoelectricity of PVDF, the focus of this part of the work was to study the effect of the ratio and content of the conductive filler reduced graphene oxide (RGO) and polypyrrole (PPY) in the matrix material polyurethane (PU) on the conductivity of electrospun nanofibers, where electrostatic repulsive forces are generated between the PPY and the RGO nanosheets, which prevents them from re-stacking and contributes to the homogeneous dispersion of the two, thus increasing the electrical conductivity of the conductive nanofiber [21]. Firstly, the effect of the ratio of PPY to RGO on the conductivity was analyzed; the conductivity of PPY was negligible compared to RGO, and the role of PPY was focused on dispersing the RGO more uniformly, so the content of RGO was fixed and the content of PPY was used as a variable in this study. Different ratios of PPY and RGO were added to DMF and dispersed by ultrasonication for 1 h. PU of 13 wt.% was dissolved in ultrasonically dispersed RGO/PPY/DMF, stirred for 2 h at 50 °C until the solution was homogeneous, and then dispersed by ultrasonication for 1 h. The solution was allowed to stand for 20 min. When the ratio of PPY to RGO was determined, the effect of the content of both compounds on the conductivity of the nanofiber membrane was studied, i.e., the mass fraction of both was varied at a fixed ratio. The above operation of solution configuration was repeated for electrospinning.

#### 2.2.3. Preparation of Piezoelectric–Conductive Composite Polymer Nanofiber Materials

The PVDF and PU/RGO/PPY solutions were loaded into 10 mL syringes and the piezoelectric–conductive composites were prepared by loading PVDF and PU/RGO/PPY solutions into 10 mL syringes under the control of a syringe pump. A constant volume flow rate of 0.5 mL/h was maintained for the PVDF solution, and a constant volume flow rate of 1.0 mL/h was maintained for the PU/RGO/PPY solution. A high voltage of 15 kV was applied when the solution was drawn into the fibers. The fibers were collected by aluminum foil covered with a roller device (speed 500 r/min) at a distance of 15 cm from the nozzle tip.

**Figure 1:**
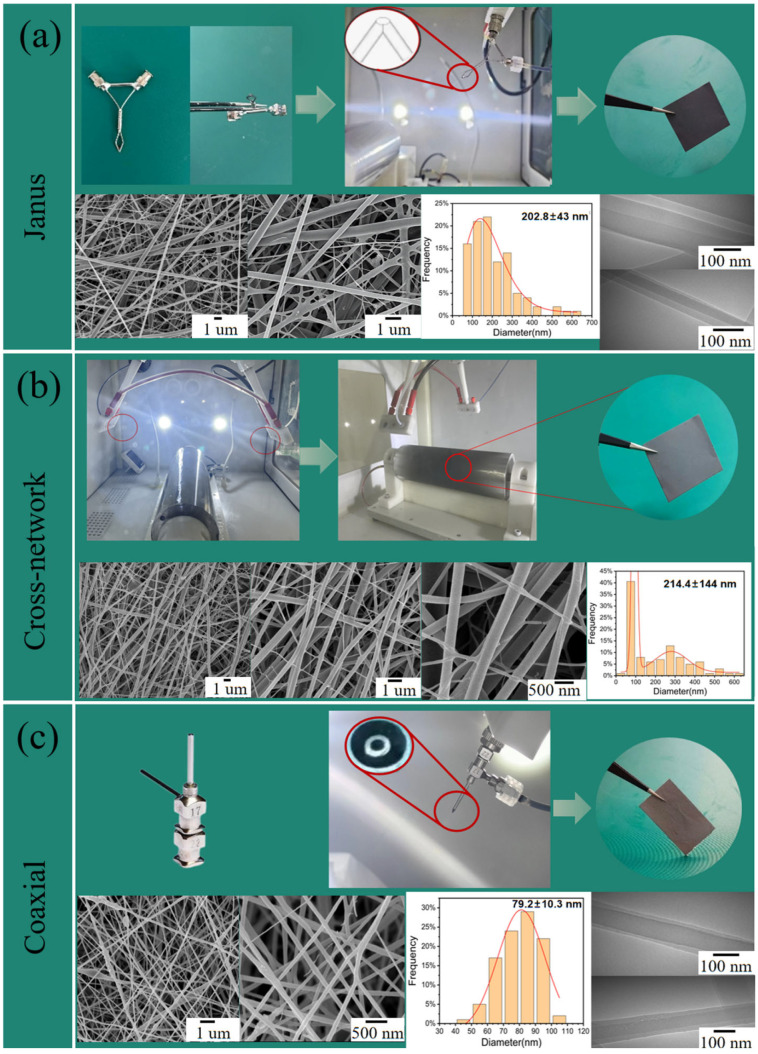
Preparation process of three piezoelectric–conductive composite nanofiber membranes with different structures and their macro–micro morphology: (**a**) Janus; (**b**) cross-network; and (**c**) coaxial.

During electrospinning, a special nozzle (22G-22G nozzles of Janus structure) was used after modification. As shown in Figure 1a, the nanofiber with a Janus structure can be prepared by using this nozzle. The composite nanofiber materials with a common cross-network structure and coaxial structure were constructed as a control. Among them, the piezoelectric–conductive composite nanofibers with a cross-network structure were prepared by using two 22G nozzles symmetrically distributed along the drum axis to spray the piezoelectric-phase PVDF and the conductive-phase PU/RGO/PPY, respectively, and the two jets were intertwined together to form a composite material under the rotation of the drum receiver. The preparation method of piezoelectric–conductive composite nanofibrous materials with the cross-network structure is shown in Figure 1b. The preparation of coaxially structured nanofibers requires the use of a special coaxial nozzle. Coaxial-structured piezoelectric–conductive composite nanofibers are single fibers that exhibit a shell–core structure. The coaxial structure has strict requirements for the shell-to-core flow rate ratio and total flow rate, and in this study, the flow rates of both the core PVDF and shell PU/RGO/PPY could not reach the optimal level (flow rate of 0.2 mL/h for PVDF and 0.7 mL/h for PU/RGO/PPY). For the preparation of coaxially structured nanofibers, 22G-17G nozzles with coaxial structures were used; the nozzle structure and preparation process of the coaxial-structured composites are shown in Figure 1c.

### 2.3. Characterization

The microscopic morphology of the sample was observed by scanning electron microscopy (SEM, Hitachi Regulus-8220, Hitachi, Tokyo, Japan). Fourier transform infrared spectra (FTIR) were obtained on an FTIR spectrometer (BRUKER VERTEX 70 RAMI, BRUKER, Billerica, MA, USA). The porosity and pore size of the samples were evaluated by mercury intrusion porosimetry (MIP) on a fully automatic mercury piezometer (Micromeritics AutoPore V 9620, Micromeritics, Atlanta, GA, USA). The Janus and coaxial structures of electroactive nanofibers were observed by transmission electron microscopy (TEM, JEM-2100, JEOL, Tokyo, Japan). Mechanical properties of the samples were tested by a universal testing machine (CT5105 100 KN, MTS, Eden Prairie, MN, USA). The specimen was cut into dumbbell sections of size 10 × 50 mm, and a minimum of five samples were tested. Then, samples were stretched at the speed of 1 N/min at room temperature and the stress–strain curves, tensile stress, and elongation at break were determined. Wettability of the samples was evaluated by the water contact angle measurement equipment (OCA25, Dataphysics, Stuttgart, Germany), and six parallel tests were carried out on the different areas of each sample. The conductivity of the electroactive outer layer was tested with a multifunctional digital four-probe tester (Cryoall CTA-3, Beijing Kerio Technology Co., Beijing, China). The piezoelectric constant d33 of the electroactive layer of the sample was analyzed by a quasi-static d33 meter (ZJ-3A, Institute of Acoustics, Chinese Academy of Sciences, Beijing, China).

### 2.4. In Vitro Osteogenic Differentiation Assessment

The cell morphology and adhesion of cells on the surface of piezoelectric–conductive polymer nanofiber materials with different structures were observed by in vitro cell experiments to explore the cytocompatibility of each sample. The samples were co-cultured with mouse embryonic osteoblast precursor cells (MC3T3-E1) (2 × 104 cells per well) in 24-well plates and compared with a blank control (tissue culture plastic, TCP). The seeded samples and the blank control were incubated in D-MEM (high sugar) containing 10% FBS and 2% penicillin/streptomycin, and then the samples were incubated at 37 °C in 5% CO_2_. The culture medium was changed every 2–3 days. MC3T3-E1 cells were cultured on the samples for 1, 3, 5, and 7 days, then 10 μL of CCK-8 reagent was added to each well to detect the proliferative activity of the cells cultured on the samples, and the cells were incubated in an incubator at 37 °C for 2 h. The absorbance values of the wells were detected with a multifunctional enzyme marker (BIO-RAD, BIO-RAD680, Hercules, CA, USA) at a wavelength of 450 nm. The activity of alkaline phosphatase (ALP) of MC3T3-E1 was analyzed by co-culturing for 1, 3, 5, and 7 days to assess osteogenic differentiation.

### 2.5. Statistical Analysis

All experiments were repeated three times. All data were expressed as the arithmetic mean ± standard deviation. The statistical significances were determined via the one-way analysis of variance (ANOVA) or repeated measures ANOVA in SPSS27.0 software. *p* ≤ 0.05 was considered to be statistically significant.

## 3. Results and Discussion

### 3.1. Optimization of Conductive-Phase Conductivity

**Figure 2:**
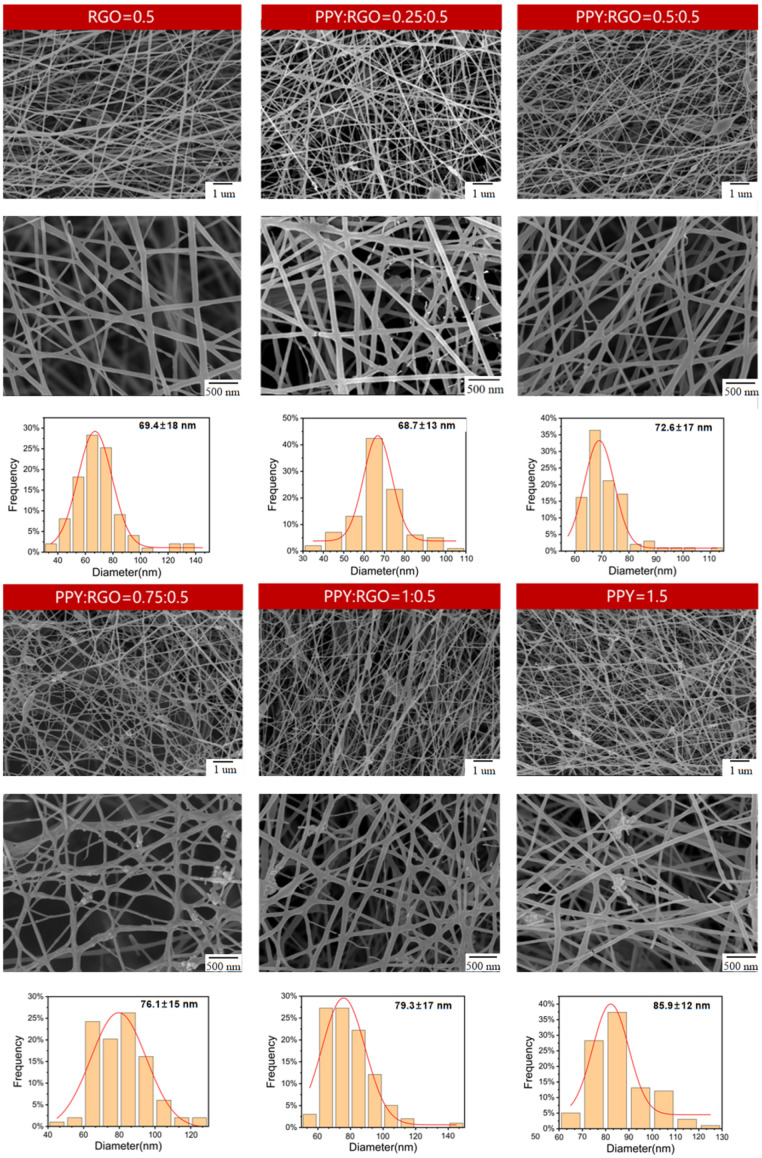
SEM images of samples PR0.5P0, PR0.5P0.25, PR0.5P0.5, PR0.5P0.75, PR0.5P1, and PR0P1.5.

The mass fraction of RGO was kept constant, whereas the mass fraction of PPY was varied, resulting in different ratios of RGO to PPY (Table 2). Six groups of conductive fiber samples were labeled as PR0.5P0, PR0.5P0.25, PR0.5P0.5, PR0.5P0.75, PR0.5P0.75, PR0.5P1, and PR0P1.5 based on their varying doping concentrations. Sample PR0P1.5 served as a control group to illustrate that nanofibers doped with a relatively high PPY content exhibited significantly lower electrical conductivity compared with sample PR0.5P0. The microscopic morphology diameter distribution of the electrospun nanofibers is depicted in Figure 2. The introduction of PPY and RGO into PU resulted in varying degrees of particulate matter accumulation in the fibers. Notably, samples PR0.5P0 and PR0.5P0.25 displayed favorable morphology with uniform distribution.

**Figure 3:**
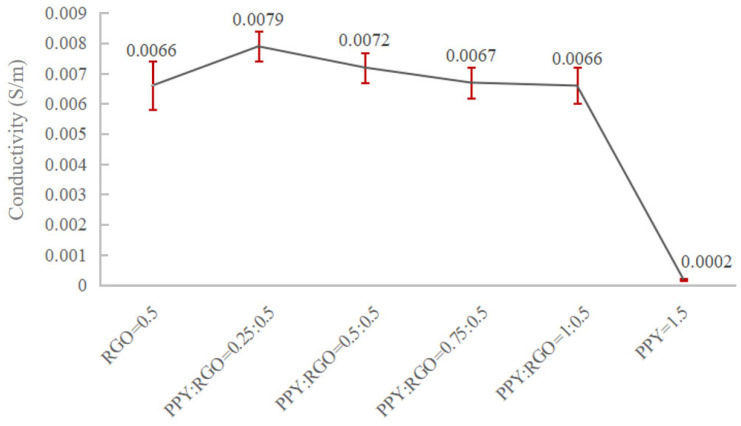
The effect of variations in the ratio of PPY to RGO on conductivity.

When the PPY content was ≥0.5 wt%, both conductive fillers, RGO and PPY, exhibited significant agglomeration, uneven fiber diameter, and more beaded polymer nanofibers. This phenomenon indicated that excessive PPY content hindered the dispersion of RGO and PPY in the fibers. Furthermore, as the PPY content increased, the agglomeration of RGO and PPY worsened, negatively impacting the preparation of conductive nanofibers. Further, the resistivity of the six samples was assessed using the four-probe method and was then converted to conductivity. The variation in conductivity of the six sets of samples is shown in Figure 3. The conductivity trend in the figure reveals that the addition of different mass fractions of PPY (i.e., different mass fraction ratios of PPY to RGO) significantly influenced the conductivity of the composite nanofibers. Specifically, the conductivity of the conductive fibers markedly increased when the PPY content was 0.25 wt%. The conductivity exhibited a decreasing trend while still retaining a positive effect when the PPY content was >0.25 wt%. It decreased when the PPY content was ≥0.5 wt%. The addition of PPY failed to positively enhance the conductivity in these cases.

Usually when the conductivity of the solution increases, the diameter of the electrospun fibers decreases, but the trend of the average diameter of the electrospun fibers in Figure 2 does not correspond exactly to the trend of the conductivity of the samples in each group in Figure 3. This is probably due to the fact that the increase in the concentration of PPY in the solution increases the viscosity of the solution, and the increase in the viscosity of the solution allows the charged jet to withstand a greater Coulomb repulsion. Among these six groups of samples, PR5P0.25 had the highest conductivity, corresponding to the smallest average fiber diameter, but the difference with PR0.5P0 was not significant because the conductivity of the two samples was close. The PR0.5P0.25 samples had greater additions of PPY, so the viscosity of the solution increased, thus offsetting part of the increase in conductivity to promote fiber stretching and resulting in the two samples having very close average diameters. Then as the concentration of PPY continued to increase, the viscosity of the spinning solution continued to increase, but the conductivity did not continue to increase (Figure 3), so the fiber diameter continued to increase. The PR0P1.5 sample had the largest average fiber diameter due to its very low conductivity and high PPY concentration.

**Figure 4:**
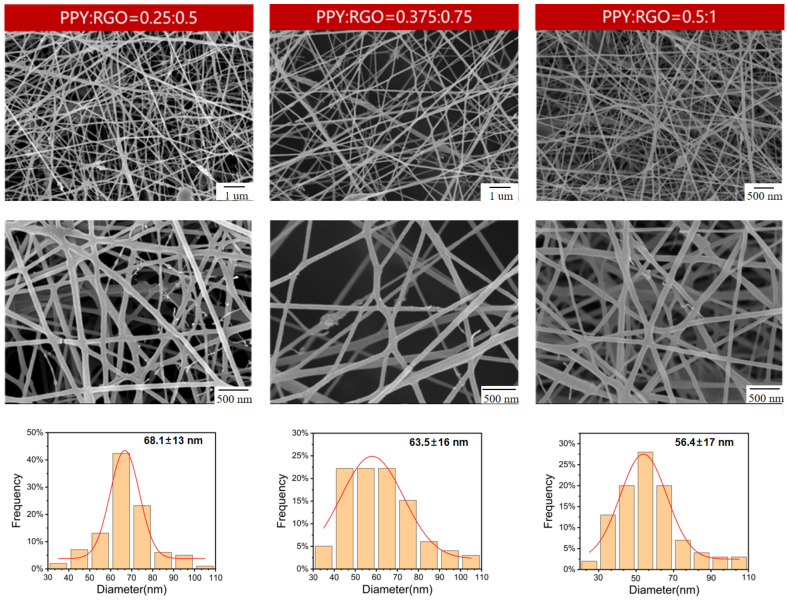
SEM images of samples PR0.5P0.25, PR0.375P0.5, and PR1P0.5.

**Figure 5:**
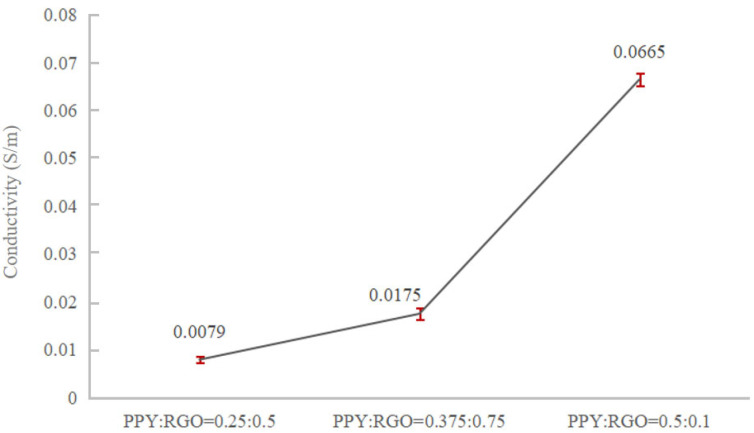
Trend of conductivity of conductive nanofibers doped with fixed ratio and different mass fractions of RGO and PPY.

**Figure 6:**
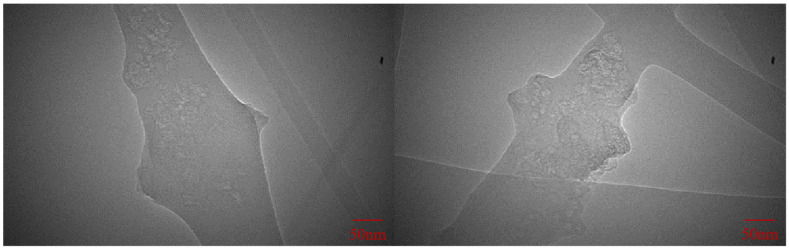
TEM of conductive fillers RGO and PPY doped in nanofibers.

Based on the aforementioned findings, the PPY to RGO ratio was established as 1:2. Then, the impact of varying contents of both substances on the conductivity of the nanofiber membrane was investigated. The experimental groups are outlined in Table 3. The samples with different PPY and RGO contents in the same ratio were labeled as PR0.5P0.25, PR0.375P0.5, PR1P0.5, and PR0.75P1.5. Notably, the spinning solution of sample PR0.75P1.5 failed to form continuous fibers due to excessive viscosity during electrospinning. As shown in Figure 4, the microscopic morphologies of PR0.5P0.25, PR0.375P0.5, and PR1P0.5 were observed and their average fiber diameters were calculated, respectively. The micrographs of the three samples were relatively similar, characterized by a uniform diameter and absence of extensive fiber breakage or spindle beading. Although the microscopic morphology alone could not fully assess the performance of the PR0.5P0.25, PR0.375P0.5, and PR1P0.5 samples, it provided further validation of the PPY to RGO ratio of 1:2. Similarly, the conductivity of these samples was determined using the four-probe method, and the change in conductivity with increasing PPY and RGO content is shown in Figure 5. With the increase in conductivity, the average diameter of the fibers decreased significantly, indicating that the increase in solution viscosity caused by an increase in the RGO and PPY content could not prevent the decrease in fiber diameter caused by the increase in conductivity. At a fixed mass fraction ratio of PPY to RGO, the conductivity exhibited an accelerated increase with higher mass fractions of PPY and RGO. Specifically, when the PPY content was 0.5 wt% and RGO was 1.0 wt%, the conductivity measured 6.65 × 10^−2^ S/m, comparable to that of natural bone (0.02–0.06 S/m) as reported by Sierpowska et al. [22,23]. Furthermore, the loading of conductive fillers in sample PR1P0.5 was examined via TEM (Figure 6), confirming the successful encapsulation of PPY and RGO within the fibers rather than a mere surface attachment. Therefore, it was confirmed that electrospun conductive nanofibers could be prepared under a PPY to RGO ratio of 1:2, with PPY of 0.5 wt% and RGO of 1.0 wt%.

### 3.2. Fabrication and Characterization of Piezoelectric–Conductive Polymer Nanofiber Materials

#### 3.2.1. Preparation and Morphology of Piezoelectric–Conductive Polymer Fibers

The macroscopic and microscopic morphologies of the Janus-structured piezoelectric–conductive composite nanofiber material are shown in Figure 1a. During fabrication, the nozzle was connected to PVDF on one side and PU/RGO/PPY on the other side. Both solutions were extruded from the tip of the nozzle to form one-dimensional Janus-structured nanofibers under the influence of an electric field, integrating piezoelectric–conductive functions within a single fiber. The demarcation line between the two electroactive materials was visible in some fibers in the SEM image. These fibers exhibited a good morphology, and the material partitioning within a single fiber was clearly observed in the TEM images. Figure 1b illustrates the macroscopic and microscopic morphologies of the piezoelectric–conductive composite nanofibrous membrane with a cross-network structure. This structure was composed of white PVDF fibers and black PU/RGO/PPY fibers, resulting in a gray composite. The SEM images revealed that fibers of different diameters intertwined, with poorer uniformity in fiber diameters compared with the Janus structure. Coaxially structured piezoelectric–conductive composite nanofibers exhibited a shell–core structure, which offered better integration but limited accessibility to the conductive material of the shell layer. The preparation of coaxially structured fibers required more stringent control compared with the cross-network and Janus structures. Specifically, the flow rate of the shell layer was three to five times that of the core layer, and the total flow rate was not too high. The shell–core flow rate and the sum of the actual maximum spinnable flow rate of the selected material were considered. Therefore, the extrusion speeds for PVDF and PU/RGO/PPY were optimized in the preparation of coaxially structured piezoelectric–conductive composite nanofibrous materials. PU/RGO/PPY can be used as the shell layer of the coaxial fibers only due to the limitation of the spinnable flow rate ranges of the spinning liquids of PVDF and PU/RGO/PPY. Further, PVDF can be used as the core layer of the coaxial fibers only. In this study, the spinnable range was 0.2–1.2 for PVDF and 0.4–1.5 for PU/RGO/PPY. The electrospinning process was most stable when the flow rate of the spinning solution was 0.2 mL/h for PVDF and 0.7 mL/h for PU/RGO/PPY. However, when the flow rates of the two solutions were not at the aforementioned values, the electrospinning process showed different degrees of filament breakage as well as continuous jittering of the Taylor cone. This indicated that the process for the coaxial-structured nanofibers was even more uncontrollable and less stable. The preparation process and macroscopic and microscopic morphologies of coaxial-structured composites are shown in Figure 1c. The coaxially structured fibers had good homogeneity of their diameters, but poor process stability resulted in spindle-shaped beads and filament breakage. TEM observations confirmed the successful preparation of the coaxial structure in the nanofibers.

#### 3.2.2. Structural Features of Piezoelectric–Conductive Nanofibers in Relation to Their Piezoelectricity and Electrical Conductivity

**Figure 7:**
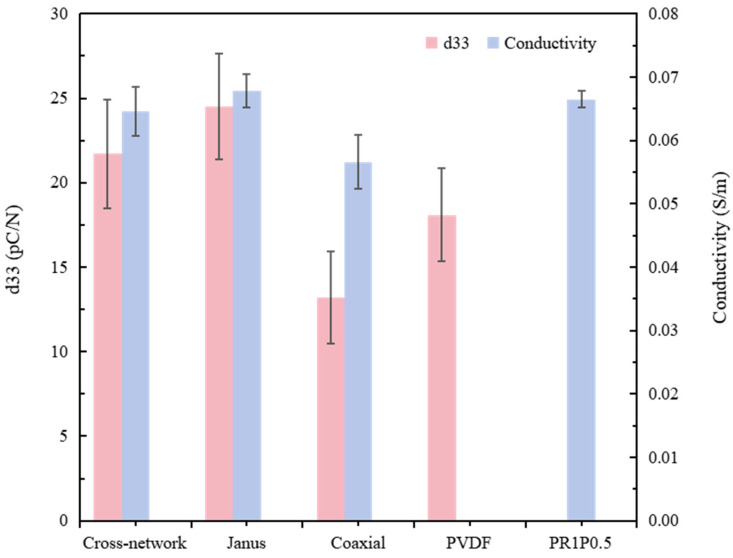
Comparative analysis of the electrical activity of three different structures of piezoelectric–conductive composite nanofiber materials.

The piezoelectric constants and electrical conductivities of nanofibers with cross-network, Janus, and coaxial structures were characterized, using PVDF fibers and PU/RGO/PPY fibers as the control group. The results are presented in Table 4 and Figure 7. The Janus structure of nanofibers exhibited the highest overall comprehensive electrical activity, primarily due to the incorporation of RGO in the conductive portion of the nanofibers. RGO consisted of closely stacked single-layered two-dimensional honeycomb structures formed by sp2-hybridized carbon atoms. These highly electronegative carbon atoms interacted electrostatically with the low-electronegativity hydrogen atoms in the piezoelectric-phase PVDF chain within the fiber. This electrostatic interaction induced the formation of the electrically active β-phase in the PVDF, enhancing the material’s piezoelectric performance. Additionally, the strong conductivity of the PU/RGO/PPY spinning solution caused the highly conductive material to be more thoroughly stretched during the electrospinning process under the electric field force. The stretching force was transferred to the piezoelectric-phase PVDF, resulting in better stretching of the composite fibers and enhancing their piezoelectric performance. Compared with pure PVDF, the piezoelectric constant increased by 34.8%. For the cross-network structure of piezoelectric–conductive nanofibers, two jets intertwined after being ejected from the nozzle during electrospinning. As they traveled from the nozzle to the collector plate, the intertwining conductive and piezoelectric phases underwent the aforementioned piezoelectric enhancement effect, increasing the piezoelectric constant of the composite fibers. However, compared with the Janus structure, the less intimate contact between the piezoelectric–conductive nanofibers in the cross-network structure resulted in a less significant enhancement effect on the piezoelectric properties. In the coaxial structure, the piezoelectric–conductive phases were not prepared with the optimal process parameters, which adversely affected the electrospinning process. Then, the piezoelectricity and conductivity of the composite nanofibers were lower than those of the control groups of PVDF and PU/RGO/PPY, respectively.

Based on the analysis of the properties of piezoelectric–conductive materials, as well as the experimental processes and results, the relevant theories of piezoelectric–conductive composites based on structural design are summarized as follows. These theories can be generalized and applied to piezoelectric–conductive composites with various preparation processes and structures:

(1) Independent electroactive unit composite.

In the case of both piezoelectric–conductive materials, the composite process must maintain their respective piezoelectric–conductive properties. These properties persist as independent piezoelectric units and independent conductive units within the composite. Hence, the composite material can simultaneously exhibit piezoelectricity and conductivity, which are known as self-generated conductive electrically active materials.

(2) Single electroactivity of piezoelectric–conductive materials upon mutual doping.

**Figure 8:**
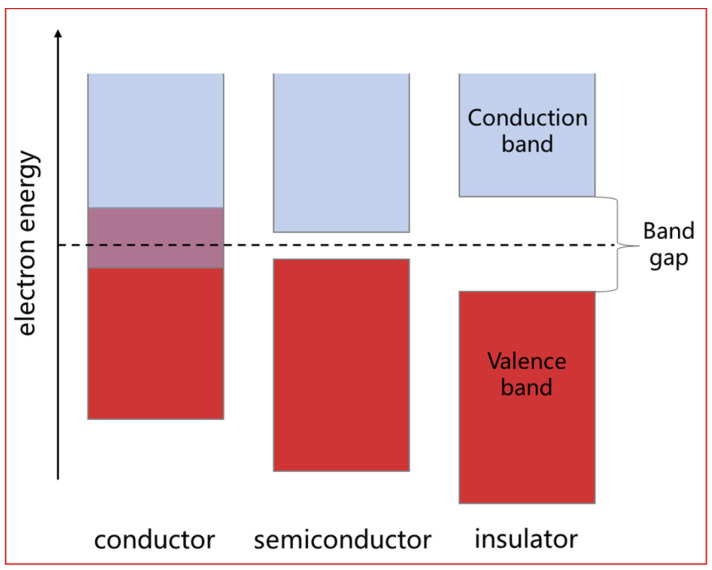
Electronic energy bands of different types of materials. (The red part of the figure is the valence band and the blue part is the conduction band).

When piezoelectric–conductive materials are combined, one electroactive material is used as the matrix and the other as the dopant. The composite material exhibits at most a single electroactive property. This phenomenon can be explained by the inherent nature of materials. In a solid material, specific energy levels exist for electrons, forming distinct energy bands. Electrons primarily occupy the lowest energy band, known as the valence band. For conductive materials, an overlapping energy band called the conduction band exists, where electrons move freely. In contrast, insulators, including piezoelectric materials, have a large energy band gap between the conduction and valence bands, preventing electrons from crossing (Figure 8) [24]. Piezoelectric materials act as insulators when used as dielectrics and are mechanically stimulated to change the distribution of positive and negative charges within the material, resulting in the generation of an electric field. The addition of conductive particles to the composite material increases the number of freely movable electrons and enhances their mobility. When the content of conductive particles falls within a certain range, they are distributed among numerous insulating materials with spaces between them, hindering the continuous movement of electrons. At this point, the piezoelectric enhancement effect is observed. However, as the content of conductive particles increases, the overall amount of freely movable charge gradually increases, enhancing the mobility and migration ability of electrons. Under the influence of the polarized electric field, electrons in the valence band acquire sufficient energy to cross the energy band gap. This disrupts the internal equilibrium state of the electrostatic field, leading to the generation of a current and completing the transformation of the piezoelectric material into a conductive material (non-insulating material).

(3) Preferential polarization of piezoelectric materials.

It is preferable to polarize the piezoelectric material before composite formation to prevent the negative impact of the conductive filler on the piezoelectricity and conductivity of the composite. If polarization occurs after composite formation, the piezoelectric material content may lead to composite breakdown if too high, or poor conductivity if too low. Balancing these factors poses a significant challenge in material selection and preparation processes. In this study, the piezoelectric–conductive composites were prepared by electrospinning. Electrospinning offers advantages in composite preparation as it allows for simultaneous fiber curing, structure compositing, and material polarization.

#### 3.2.3. FTIR of Piezoelectric–Conductive Nanofibers

**Figure 9:**
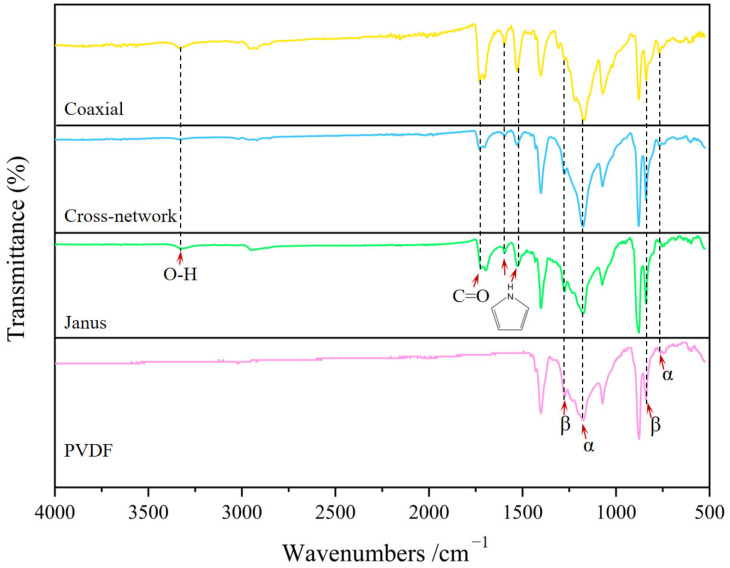
FTIR of piezoelectric–conductive nanofibers with three different structures and PVDF nanofibers.

The functional composition of the piezoelectric–conductive polymer nanofiber materials was characterized using the FTIR method. Figure 9 shows the FTIR spectra of the samples ranging from 500 to 4000 cm^−1^. Characteristic peaks corresponding to the β-phase of PVDF were observed at 840 and 1284 cm^−1^, whereas the representative characteristic peaks corresponding to the α-phase of PVDF appeared at 763 and 1182 cm^−1^ [25,26]. The β-phase characteristic peaks of the coaxial group were notably weaker, whereas those of the samples with Janus and cross-network structures exhibited more pronounced peaks compared with the PVDF. Using the Lamb–Beer law, the β-phase contents of the four groups of materials were calculated using Equation (1). The β-phase contents of the Janus group, cross-network group, coaxial group, and PVDF control group were determined to be 88.9%, 86.9%, 69.4%, and 81.8%, respectively. In summary, the findings presented in Figure 9 align with the results depicted in Table 4 and Figure 7 regarding the electroactivity of the materials [27,28]. In addition, the FTIR spectra of the piezoelectric–conductive polymer nanofiber materials revealed characteristic peaks of PPY (1501 and 1585 cm^−1^ for pyrrole ring skeletal vibrations) and RGO (1725 cm^−1^ for C=O stretching and weakened O–H stretching after a reduction reaction at 3408 cm^−1^), indicating the successful introduction of RGO and PPY into the three samples [13,29].

(1) $$F_{\beta }=\frac{A_{\beta }}{\left(\frac{K_{\beta }}{K_{\alpha }}\right)A_{\alpha }+A_{\beta }}$$

where F(β) represents the β-phase content; A_α_ and A_β_ represent the absorbance at 766 and 840 cm^−1^; and K_α_ and K_β_ are the absorption coefficients at the respective wavenumbers, with values of 6.1 × 10^4^ and 7.7 × 10^4^ cm^2^ mol^−1^, respectively.

#### 3.2.4. Water Contact Angle Analysis of Piezoelectric–Conductive Nanofibers

**Figure 10:**
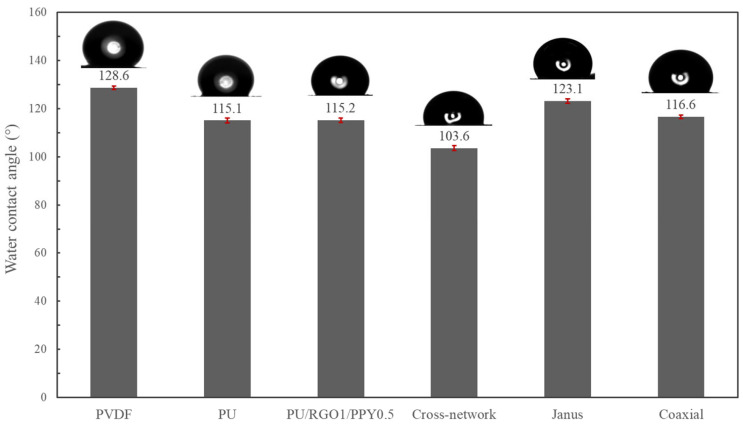
Water contact angles of three different structures of piezoelectric–conductive nanofiber materials and control groups PVDF, PU, PU/RGO1/PPY0.5.

The water contact angles of three structures of piezoelectric–conductive composite nanofiber membranes were measured (the values were taken after the water droplets fell onto the surface of each sample for 10 s), and the test results are shown in Figure 10. PVDF, PU, and PU/RGO/PPY were the control groups, and the water contact angles of PU/PPY/RGO were almost the same as that of PU, indicating that RGO/PPY was mainly incorporated into the fibers in a wrapped form with PU. PU/PPY/RGO, as the shell layer of the coaxially structured composite fibers, should have similar contact angles with PU and PU/PPY/RGO, and it was found that the contact angles of the former and the latter two differed only by about 1.5°. The cross-network structure of the nanofibers had a high degree of inhomogeneity in diameter, and this highly inhomogeneous diameter fiber interlacing may have formed some disordered physical cues on the surface of the material, making the surface of the material relatively rough. The water contact angle of Janus-structured nanofibers was intermediate between that of PVDF and PU/PU/PPY/PROY, and based on the structural characterization of the Janus nanofibers, this result is reasonable and obvious. All three structured piezoelectric–conductive composites are hydrophobic, and hydrophobic materials can exhibit antimicrobial properties to some extent and are not favorable for fibroblast adhesion. The JANUS structure nanofiber material showed better hydrophobicity compared to the other two structures of nanofiber material [30,31,32].

#### 3.2.5. Pore Structure Analysis of Piezoelectric–Conductive Nanofibers

**Figure 11:**
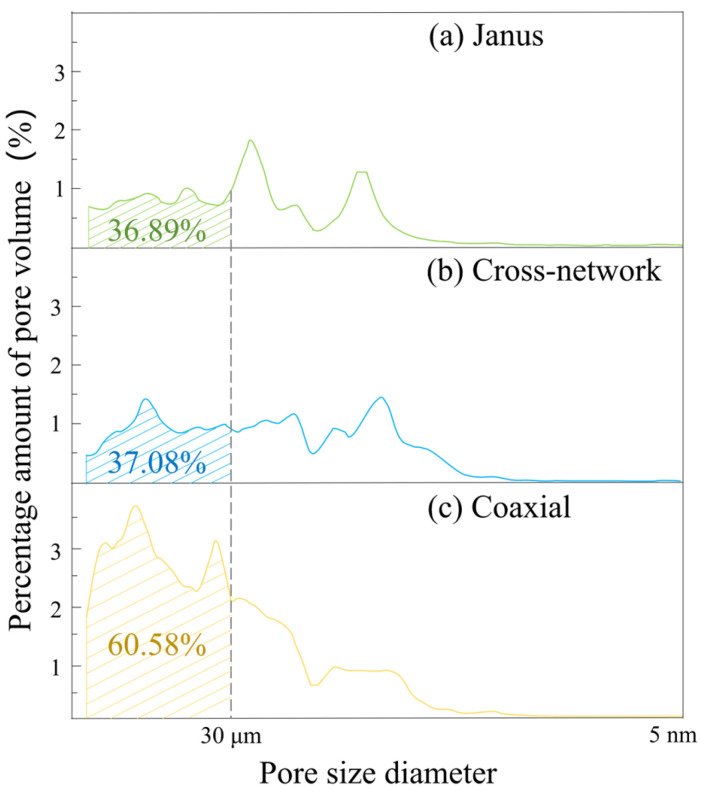
Pore volume distribution curves corresponding to the pore size of three piezoelectric–conductive nanofiber materials: (**a**) Janus; (**b**) cross-network and (**c**) coaxial.

The pore size and porosity of three different structures of piezoelectric–conductive composites were characterized by MIP, and the information of the main pore structure of the three structures is shown in Table 5 and Figure 11. Figure 11 shows a distribution curve of pore volume percentage for corresponding pore sizes, where the horizontal coordinate of the graph represents the pore size and the vertical coordinate represents the percentage of pore volume with respect to the corresponding pore size. The porosity of the fibrous materials of all three structures was ≥70% (high porosity is a great natural advantage of electrospinning materials), which is larger than some of the materials that have been reported to be used for bone tissue engineering [33,34,35,36,37]. The diameter of fibroblasts was in the range of 20–30 μm, and the percentage of pore volume of coaxial structure nanofibers with a pore diameter greater than 30 μm was 60.58%, while those of the Janus and cross-network structures were 36.89% and 37.08%, respectively. It can be seen that the pore structure of the coaxially structured nanofiber membrane is mainly dominated by large pore diameters. The average pore size of the cross-network structure, Janus structure, and coaxial structure of the fiber material was 2.26 μm, 1.19 μm and 6.95 μm, respectively. The nanofibrous materials with a Janus structure possessed smaller pore sizes and fewer macroporous pores, which were more advantageous in preventing fibroblasts from invading the bone tissue at the damaged place. If the Janus-structured piezoelectric–conductive nanofiber material is covered on the surface of the bone defect, it can show a better ability to block the invasion of fibroblasts without affecting nutrient transport and exchange.

#### 3.2.6. Mechanical Properties of Piezoelectric–Conductive Nanofibers

**Figure 12:**
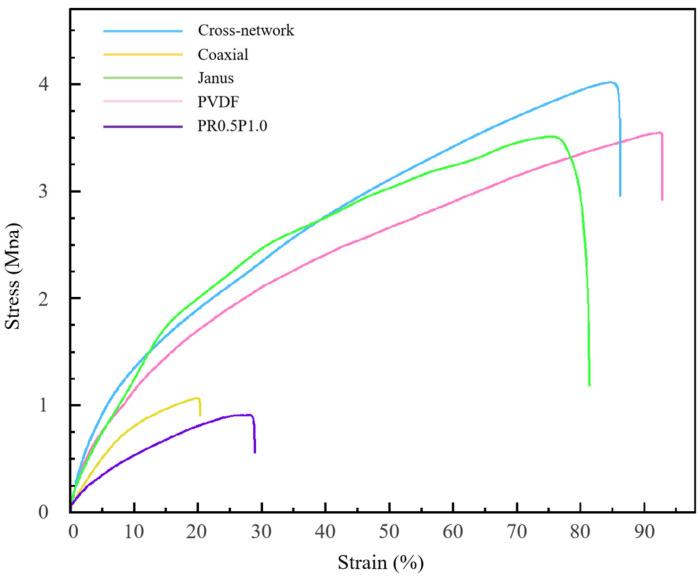
Stress–strain curves of three different structures of piezoelectric–conductive nanofibers, PVDF piezoelectric nanofibers, and PR0.5P1.0 conductive nanofibers.

The mechanical properties of nanofiber materials with different structures are depicted in Figure 12. The tensile strengths of the cross-network structure, Janus structure, and coaxial structure nanofiber materials were 4.12 ± 1.04, 3.59 ± 0.69, and 1.15 ± 0.47 MPa, respectively. In contrast, the tensile strengths of the control groups, PVDF and PR1P0.5, were 3.62 ± 1.12 and 0.91 ± 0.52 MPa, respectively. The cross-network structure sample, where piezoelectric-phase fibers and conductive-phase fibers were interwoven, exhibited minimal impact on the mechanical properties of the piezoelectric-phase PVDF fibers. The tensile strength and elongation at the break of this sample are primarily determined by the piezoelectric-phase PVDF, resulting in similar mechanical properties to those of the PVDF control group. However, the slightly higher tensile strength of the cross-network structure sample may be attributed to the enhancement of intermolecular forces due to the presence of conductive nanoparticles on the surface of some fibers. Similarly, the Janus structure group demonstrated mechanical properties comparable to those of the PVDF control group. However, the piezoelectric phase underwent more significant stretching during electrospinning due to the closer contact between the piezoelectric and conductive phases in the Janus structure. Therefore, the maximum tensile strength of Janus-structured nanofibers was lower than that of the cross-network-structured nanofibers but still close to that of the control PVDF. This might be attributed to the conductive nanoparticles promoting the crystallinity of the piezoelectric-phase PVDF and maintaining the stability of the Janus-structured nanofibers. Note that the crystallization-assisting components such as nanoparticles, organic salts, inorganic salts, and so on, along with a stronger tensile force, were beneficial for increasing the crystallinity of PVDF. On the contrary, the polar groups in the conductive nanoparticles were attracted to the highly polar F atoms in the piezoelectric-phase PVDF, forming a three-dimensional structure of the fibers with multiple linkages. This also helped maintain the stability of the piezoelectric-phase PVDF and improve the mechanical properties of the Janus-structured nanofibers. However, the presence of the conductive phase in the Janus structure nanofibers resulted in a lower elongation at the break compared with the PVDF control group. The conductive fiber PR1P0.5 exhibited increased brittleness and reduced toughness due to the introduction of conductive particles in the PU. Moreover, the samples with a coaxial structure displayed the poorest mechanical properties due to suboptimal process parameters. Although their tensile strength was marginally higher than that of the control PR1P0.5, the elongation at the break was significantly lower. A material must possess sufficient strength to withstand the stresses exerted by surgical devices and dynamic tissues during physiological activities to ensure successful clinical application. In our study, the samples with Janus and cross-network structures demonstrated stretchability, which is essential for bone implant materials, especially artificial periosteum materials. The tensile strengths of the samples with both structures were around 4 MPa, which is similar to that of the natural periosteum (3–4 MPa), fulfilling basic clinical requirements [38].

### 3.3. In Vitro Cytocompatibility Assay

#### 3.3.1. CCK-8 Assay to Detect Cell Proliferation

The proliferation of MC3T3-E1 cells on different structures of piezoelectric–conductive composite polymer nanofiber materials was evaluated using the CCK-8 assay. When co-cultured with three types of piezoelectric–conductive composite polymer nanofiber materials, MC3T3-E1 cells were subjected to repeated measurements of optical density (OD) values at 1, 3, 5, and 7 days. The results of the variance analysis are presented in Table 6. Differences in cell proliferation at different time points are statistically significant (F = 344.111, *p* < 0.001). Similarly, significant statistical differences in cell proliferation are observed among different structures of piezoelectric–conductive composite polymer nanofiber materials (F = 154.163, *p* < 0.001). Furthermore, there is a significant interaction between time and grouping factors (F = 8.226, *p* < 0.001), indicating that data from different groups vary with time. At 1 day of co-culturing, compared to the control group, both the cross-network structure group and Janus structure group exhibited higher cell proliferation activity, while the coaxial structure group showed lower cell proliferation activity than the control group; the differences in OD values were statistically significant (*p* < 0.05). During the 3rd, 5th, and 7th days of culture, the cell proliferation activity on the three groups of piezoelectric–conductive composite polymer nanofiber materials gradually increased. When compared to the control group, the differences in the OD values were statistically significant (*p* < 0.05). This indicates that regardless of the specific day, the structural differences of the materials had a significant impact on cell proliferation. Similarly, when co-cultured with cells, irrespective of the material structure, there is a noticeable enhancement in the cell proliferation rate as the culture time increases. This indicates that once the material structure for co-culturing with cells is determined, time becomes an important factor influencing cell proliferation.

**Figure 13:**
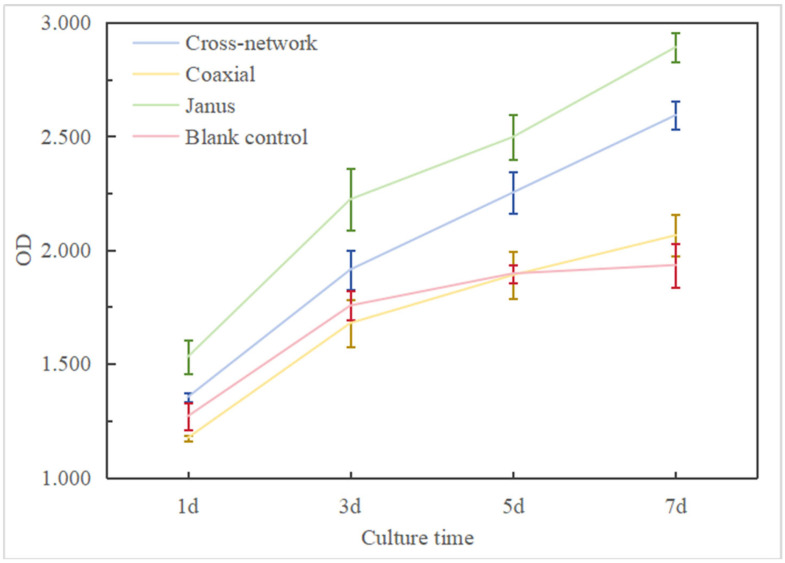
Cell proliferation images of MC3T3-E1 co-cultured with piezoelectric–conductive nanofiber materials in each group.

**Figure 14:**
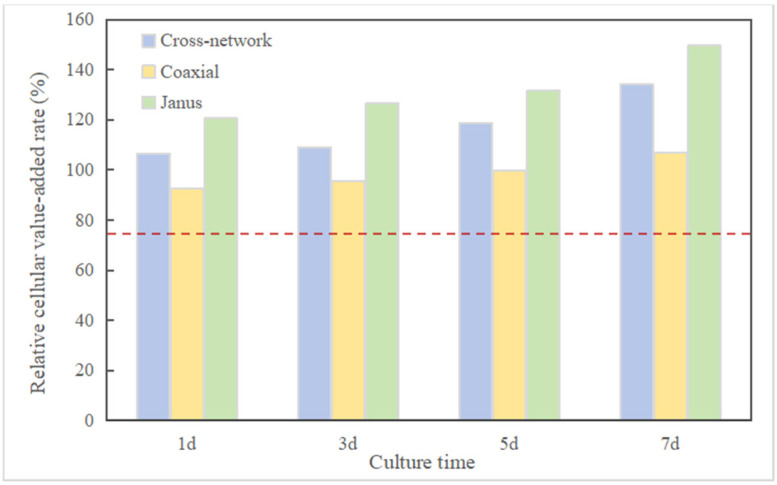
Relative cell proliferation rate of piezoelectric–conductive nanofiber materials in co-culture with MC3T3-E1. (The red line indicates a relative proliferation rate of 75% and no cytotoxicity above this line).

As shown in Figure 13, both the cross-network group and the Janus group exhibited higher cell proliferation activity than the coaxial group and the control group at these four time points. Furthermore, the cell proliferation activity of the Janus group surpassed that of the cross-network group at all four time points. Prior to the fifth day, the cell proliferation activity of the coaxial group was lower than that of the control group. By the fifth day, the cell proliferation activities of the two were nearly equal. By the seventh day, the cell proliferation activity of the coaxial group had exceeded that of the control group. The three experimental groups all had a certain promoting effect on cell proliferation, and the characterization results of their electroactivity to some extent reflect the positive influence of electroactivity on cell proliferation. The calculated relative proliferation rates of the MC3T3-E1 cells for all experimental groups were ≥90% (with the cross-network group and Janus group both ≥100%). The cytotoxicity level of the coaxial group gradually decreased from level 1 to level 0, while the cytotoxicity of the cross-network group and Janus group remained at level 0 from the beginning, and according to the cytotoxicity level standard table provided, it can be inferred that the three piezoelectric–conductive composite polymer nanofiber materials exhibit no cytotoxicity, thereby demonstrating excellent cell compatibility, as shown in Table 7 and Table 8 and Figure 14.

#### 3.3.2. Alkaline Phosphatase Activity of Cells on Piezoelectric–Conductive Nanofibers

**Figure 15:**
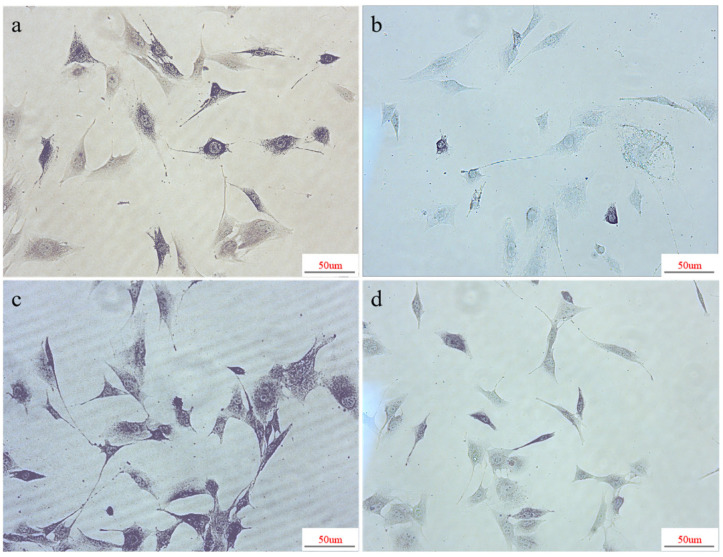
Alkaline phosphatase staining of MC3T3-E1 and piezoelectric–conductive nanofiber materials after 7 days of co-culture: (**a**) cross-network; (**b**) coaxial; (**c**) Janus; and (**d**) blank control.

The activity of alkaline phosphatase (ALP) can serve as a biochemical and histological indicator for the early evaluation of bone formation. Alkaline phosphatase staining allows for the qualitative observation of ALP production, wherein a darker staining on the material surface indicates higher ALP secretion. When co-cultured with the three piezoelectric–conductive composite polymer nanofiber materials for 7 days, MC3T3-E1 cells exhibited more pronounced ALP staining than the control group under microscopic observation, suggesting elevated alkaline phosphatase activity. This indicated the presence of a greater quantity of enzyme molecules in the samples, or a faster reaction rate of the enzyme molecules, reflecting a higher metabolic activity or functional status of the cells or tissues. The ALP staining of the Janus experimental group (Figure 15c) was significantly stronger than that of the cross-network experimental group (Figure 15a) and the coaxial experimental group (Figure 15b). The ALP staining of the cross-network experimental group was more pronounced compared with the coaxial experimental group, whereas the ALP staining of the coaxial experimental group was lighter than the other two experimental groups and was closer to that of the blank control group. Considering the increasing trend in the cell proliferation rate of the coaxial group, a stronger ALP staining than the control group may be observed in the later stages.

## 4. Conclusions

In this study, PVDF was selected as the piezoelectric phase, with 1 wt% RGO and 0.5 wt% PPY incorporated into PU as the conductive phase. Piezoelectric–conductive composite nanofibers with Janus, cross-network, and coaxial structures were fabricated via electrospinning at a voltage of 15 kV, and all three structures demonstrated successful integration of piezoelectricity and conductivity. Among these, Janus-structured nanofibers exhibited the most pronounced electroactivity. The Janus structure, with RGO and PPY acting as both piezoelectric enhancers and conductive phases, significantly enhanced the piezoelectricity of the composite fibers, resulting in higher piezoelectric constants compared with control PVDF (d33 = 24.5 pC/N), while still maintaining comparable conductivity to control PR1P0.5 (conductivity = 6.78 × 10^−2^ S/m). The porous structure of Janus-structured nanofibers acted as a barrier against fibroblast invasion, with a porosity exceeding 70% that facilitated substance transport and exchange. In addition, Janus-structured nanofibers exhibited a tensile strength of 3.59 ± 0.69 MPa and a cellular accretion rate of 149.52% after 7 days. Considering the ALP results, Janus electroactive nanofibers showed promise as suitable scaffolds and periosteum materials for the repair of bone defects.

## Figures and Tables

**Table 2:** Six experimental groups with different mass fraction ratios of PPY and RGO.

PPY wt%	0	0.25	0.5	0.75	1	1.5
RGO wt%	0.5	0.5	0.5	0.5	0.5	0

**Table 3:** The mass fractions of PPY and RGO at a fixed ratio.

**PPY wt%**	0.25	0.375	0.5	0.75
**RGO wt%**	0.5	0.75	1.0	1.5

**Table 4:** Piezoelectric constant and conductivity data for three structured piezoelectric–conductive nanofiber materials and two controls.

	Cross-Network	Janus	Coaxial	PVDF	PR1P0.5
d33 (pC/N)	21.70 ± 3.20	24.50 ± 3.15	13.20 ± 2.71	18.10 ± 2.75	0
Conductivity (S/m)	(6.46 ± 0.39) × 10^−2^	(6.78 ± 0.26) × 10^−2^	(5.66 ± 0.43) × 10^−2^	0	(6.65 ± 0.13) × 10^−2^

**Table 5:** Major pore structure data for three structures of piezoelectric–conductive nanofiber materials.

	Janus	Cross-Network	Coaxial
Pore diameter (nm)	1194.36	2264.46	6947.28
Porosity	70.09%	71.10%	78.96%
Total percentage of pore volume with pore diameter ≥30 μm	36.89%	37.08%	60.58%

**Table 6:** Proliferation of MC3T3-E1 in co-culture with piezoelectric–conductive nanofiber materials of each group.

Group	Time (day)				F	*p*
	1d	3d	5d	7d		
Cross-network	1.353 ± 0.020	1.914 ± 0.087	2.253 ± 0.090	2.593 ± 0.063	166.385	<0.001
Coaxial	1.173 ± 0.012	1.679 ± 0.105	1.890 ± 0.105	2.065 ± 0.090	58.004	<0.001
Janus	1.530 ± 0.075	2.223 ± 0.137	2.497 ± 0.097	2.892 ± 0.064	103.718	<0.001
Blank control	1.269 ± 0.059	1.756 ± 0.064	1.897 ± 0.040	1.934 ± 0.096	61.025	<0.001
F	28.187	16.595	34.626	95.178		
*p*	<0.001	<0.001	<0.001	<0.001		
Time main effect					344.111	<0.001
Group main effect					154.163	<0.001
Time × Group					8.226	<0.001

**Table 7:** Cytotoxicity grading criteria.

Toxicity Degrees	Relative Proliferation Rate (%)	Cytotoxicity Evaluation
0	≥100	Non-cytotoxic
1	75–99	Non-cytotoxic
2	50–74	Mild cytotoxicity
3	25–49	Moderate cytotoxicity
4	1–24	Moderate cytotoxicity
5	0	Severe cytotoxicity

**Table 8:** Relative value-added rates and toxicity grades of MC3T3-E1.

Group	Time (day)							
	1d		3d		5d		7d	
	Relative Proliferation Rate (%)	Toxicity Degrees	Relative Proliferation Rate	Toxicity Degrees	Relative Proliferation Rate	Toxicity Degrees	Relative Proliferation Rate	Toxicity Degrees
Cross-network	106.09	0	109.02	0	118.77	0	134.07	0
Coaxial	92.46	1	95.63	1	99.61	1	106.77	0
Janus	120.54	0	126.61	0	131.65	0	149.52	0

## Data Availability

Data are available upon reasonable request.
